# Methods for studying health disparities in U.S. nursing homes: a scoping review

**DOI:** 10.1186/s12913-025-13071-3

**Published:** 2025-08-20

**Authors:** Hanne Marie Rostad, Lucille Xiang, Elizabeth M. White

**Affiliations:** 1 Center for Care Research East, Norwegian University of Science and Technology, Gjøvik, Norway; 2 Center for Gerontology and Healthcare Research, Brown University School of Public Health, Providence, USA

**Keywords:** Health equity, Health disparities, Nursing homes, Long-term care, Review

## Abstract

**Background:**

Health disparities exist across healthcare settings, including nursing homes, contributing to preventable differences in care quality. Health disparities are a global issue, yet most studies on nursing home health disparities have been conducted in the United States. In this scoping review, our objective was to synthesize methods used in U.S. nursing home disparities research to gain insights to inform similar research in other countries. Specifically, we summarized different approaches for conceptualizing and measuring health disparities, available data sources, study designs, and analytic strategies.

**Methods:**

We employed two parallel search strategies across five databases, targeting specific aspects of health disparities and broader concepts. Study selection was conducted independently by two reviewers. Using a numerical and analytic approach, we categorized and summarized the results.

**Results:**

The search yielded 6,817 records, with 82 unique studies meeting the inclusion criteria. All studies used quantitative methods, with only two incorporating mixed methods. Most were observational cross-sectional studies (*n* = 60), while 21 were longitudinal studies, and 1 was a randomized controlled trial. Most studies used administrative data (*n* = 62). The majority (*n* = 65) measured differences in health outcomes across nursing homes. A significant number of studies (*n* = 71) focused on racial and/or ethnic health disparities, and a few studied clinical conditions (*n* = 7), rural–urban location of the nursing home (*n* = 4), socioeconomic factors (*n* = 4), age (*n* = 1), and sex (*n* = 1) as characteristics to measure disparity. Outcomes were grouped into five domains: 1) Quality of care measures (*n* = 54), 2) Infection and infection prevention (*n* = 22), 3) Transitions and acute care utilization (*n* = 19), 4) Behavioral and mental health (*n* = 18), and 5) Palliative care, end-of-life and death (*n* = 13). Across the five domains, the most prevalent outcome category studied was ‘Hospitalization and emergency room use’ (*n* = 15).

**Conclusion:**

This review highlights key issues for future research on health disparities in nursing homes, including the need to: 1) clarify concepts of health disparities and health equity; 2) move beyond mere descriptions of disparities to identify underlying factors contributing to those disparities; 3) broaden examination of disparities beyond a single axis such as race or sex; 4) integrate more qualitative data to capture nuances that cannot be measured from quantitative data; and 5) specify whether within or across nursing home differences are studied.

**Supplementary Information:**

The online version contains supplementary material available at 10.1186/s12913-025-13071-3.

## Background

Nursing homes are an important site of post-acute and long-term care for older adults with complex medical, social, and functional needs. While nursing home care quality has been a public policy concern for decades [1–4], the COVID-19 pandemic exacerbated and drew public attention to the long-standing vulnerabilities of this sector.

Over the past two decades, a number of studies have examined health disparities in nursing homes. Health disparities may occur if certain residents are being treated differently *within* a nursing home due to discrimination or prejudice that leads to unequal treatment based on individual characteristics such as socioeconomic status, race, or ethnicity [5, 6]. Health disparities may also be found *across* nursing homes if individuals belonging to certain subpopulations or groups are systematically admitted to poorer-quality facilities. Differences in quality across nursing homes may be attributed to a variety of factors at the institutional, community, and policy levels.

The concepts of ‘health disparity’ and ‘health inequity’ are intertwined, and it is challenging to speak of one without the other. Both terms have become increasingly familiar and used in research and the public discourse, yet they are rarely explicitly defined and are sometimes used interchangeably, despite representing distinct concepts [7]. Health inequities refer to underlying systemic issues that lead to these disparities [8], such as historical and current unequal distribution of social, political, economic, and environmental resources contributing to differences in opportunities to achieve optimal health [9, 10]. In contrast, the term ‘health disparity’ is used to describe differences in health outcomes between different population groups. These disparities can be measured and often highlight the unequal burden of disease, disability, and death among specific groups [11].

There are also differences in how these concepts are understood and defined in the literature. For the concept of'health inequity', some definitions emphasize the preventable and unjust nature of differences in health, focusing on the moral imperative to address them [12], while other definitions highlight the unavoidable link between health and social determinants such as socioeconomic status, race, and geographic location, emphasizing the broader social factors that contribute to health inequities [13]. For health disparities, some definitions include a wider range of determinants and attributes affecting broader health outcomes [14], while others focus more on, e.g., quality of care and compare minority and non-minority populations with similar backgrounds [15].

Research on health disparities in nursing homes has primarily been conducted in the U.S. Understanding the strengths and limitations of the U.S. literature, specifically with regards to what study methods and data sources are used, and how key concepts are defined and measured, provides opportunities to inform research development on this topic in other countries, and also in the U.S. These insights may furthermore facilitate comparisons of healthcare systems, allowing researchers to learn from and build upon different approaches in disparities research. This cross-pollination of ideas and methods can ultimately lead to more effective strategies for tackling health disparities in long-term care worldwide.

There has been no comprehensive review of methods used to study health disparities in nursing homes. This review aims to inform and support future research by identifying the scope and topical areas of existing literature, identifying key concepts, gaps, and types of evidence to provide a foundational understanding that can guide future, more targeted research efforts. The specific objective of our scoping review is to identify and summarize methods used to study health disparities in nursing homes, including study designs, data sources, levels of analysis, covariates, primary independent variables, and outcomes.

## Methods

We used five stages of the scoping review methodological framework as described by Arksey and O'Malley [16, 17]: identifying the research question, identifying relevant studies, study selection, charting the data, and collating, summarizing, and reporting the results. The optional sixth step, ‘Consultation’, was not included in this review. We followed the PRISMA extension for scoping reviews reporting guidelines [18].

### Search strategy

A comprehensive search strategy was developed in collaboration with a research librarian. The scoping review method offers greater flexibility in search design compared to systematic reviews, which emphasize comprehensive coverage. While scoping reviews also require structured searches, they allow researchers more autonomy in selecting relevant concepts and broader criteria. Research into the concepts of health disparities, inequality, and inequity is inconsistently indexed and uses a variety of terms to describe key concepts, making it difficult to reliably identify all relevant studies [19]. Our goal was to balance a comprehensive search with the ability to easily filter out irrelevant studies. To maintain relevance (precision) and comprehensiveness (sensitivity) [20], we used two parallel search strategies. First, we searched with specific domains of health disparities such as race, gender, and socioeconomic status. Second, we searched with broader concepts such as health disparity and health inequity. The search terms and two parallel search strategies employed are outlined in Supplementary file 1. Using broad search terms in the specific strategy could have increased irrelevant hits, complicating the review process. We searched Medline (Ovid), PsycINFO, Cinahl, Web of Science, and Scopus as of February 1, 2023.

### Study selection

We included studies through a two-step process. First, titles and abstracts were screened by two reviewers (HMR and LX) independently. Studies that addressed health disparities in nursing homes and were published in peer-reviewed journals were deemed eligible for full-text review. We focused exclusively on nursing homes, including both long-term and short-term care, and excluded studies focusing solely on, or comparing, nursing homes with other settings such as assisted living or home health care. To ensure the process and conduct a blind screening, we used the Covidence software as a screening tool [21]. Subsequently, all potentially relevant articles were reviewed in full text for study inclusion by two reviewers (HMR and LX) independently. Disagreements were resolved by discussion between the two reviewers.

The initial inclusion criteria were the following: 1) original study of health disparities in nursing homes in the U.S., and 2) publication in a peer-reviewed journal. The initial inclusion criteria were reviewed and revised post hoc, in light of search results and our increased familiarity with the emerging evidence [16, 17]. The following inclusion criteria were added: 3) study data were from the year 2000 or later; 4) studies must have examined differences between groups in health outcomes and/or healthcare quality, rather than merely describing various factors associated with outcomes (e.g. differences in pressure ulcer prevalence between Black and White nursing home residents, rather than just factors associated with pressure ulcer prevalence); and 5) the outcome was related to the care received in the nursing home.

### Charting the data, summarizing, and reporting the results

Data from eligible studies were charted by the first author (HMR) using a data extraction chart developed in Covidence. The first author tested the extraction chart by charting five studies to determine whether the approach to data extraction was consistent with the study’s objectives [17], discussed with other members of the research team, and revised the chart. This process continued until a consensus about the data extraction process was reached.

Data charted included: first author, publication year, sample size (nursing home residents and/or nursing homes), whether the study included long-term and/or short-term residents (“Type of stay”), study design, data sources, primary independent variable(s), level of analysis (i.e. person-level, facility-level, or both), covariates included in the analysis, whether the study examined within and/or across nursing home differences, and outcomes. Due to the large variation in how the outcomes were formulated and described, we chose to reduce the level of detail to make the results more comprehensible and useful. This was done through a thematic analysis inspired by Braun and Clarke [22], where we examined excerpts of the text relating to our study objectives and created codes that best reflected that text. The codes that thematically fit well together lead us to create subjectively cohesive categories. For example, the outcomes “pain level”, “pain assessment”, “pain management”, and “documentation of pain” were grouped into the category “Pain (management)”. Another example: “antidepressive treatment”, “treatment of depression”, “diagnosis of depression”, and “depressive symptoms” were grouped into the category “Depression (management)”. Once the categories were established, we grouped them into broader domains. Each domain represented a higher-level construct that encompassed multiple related categories to improve readability and give a more comprehensive overview.

Using a numerical approach, we made tables and descriptive representations of the charted data relating to the study objectives [23].

## Results

**Fig. 1:**
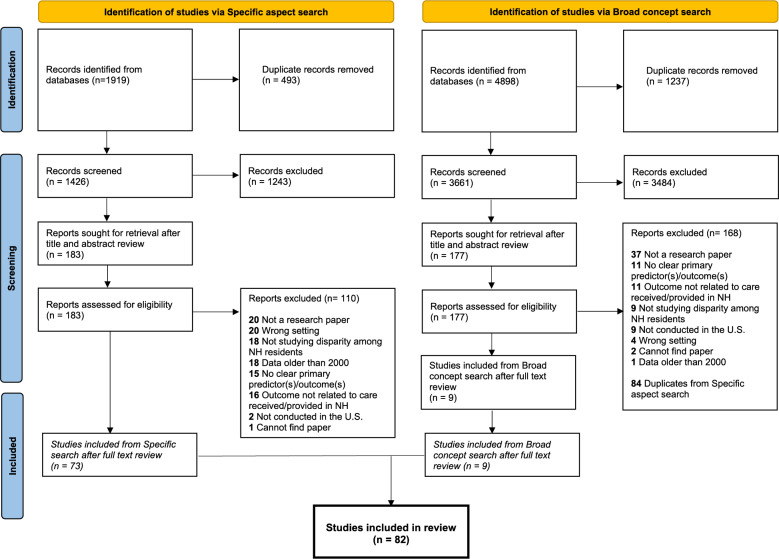
PRISMA Flow chart

Figure 1 details the selection process of studies using the two parallel search strategies, i.e., searching with 1) specific domains of health and health care disparities and 2) with broader concepts only. Starting with the specific domain search, 1919 records were retrieved. After removing duplicates, we screened the titles and abstracts of 1426 records. Of these, 183 were reviewed in full text and assessed for eligibility. A total of 73 studies were included from the specific domain search. For the broad concept search, 4898 records were retrieved. We screened titles and abstracts for 3661 records and assessed 177 full-text papers for eligibility. Of these 177 papers, 84 were duplicates from the specific search. Nine papers were uniquely identified in the broad concept search and included. In total, 82 studies were included in this review.

Table 1 summarizes the 82 studies included: 30 (37%) were very recent publications (2020–2023), 47 (57%) from the years 2010–2019, and five (6%) from the period 2000–2009. The number of facilities included in the study ranged from 1 to 17,953, while sample sizes of nursing home residents ranged from 142 to 7,734,348. Twenty-eight studies (34%) examined long-stay residents, nine (11%) studied short-stay residents, and 45 (55%) included both long- and short-stay residents.

**Table 1:** Study design and data sources

First author (publication year)	Number of facilities	Number of residents	Type of NH stay ^a^	Study design	Data sources						
					MDS	OSCAR/CASPER	Medicare claims	LTCFocus	Care Compare	AHRF	Other
Bardenheier [24]	403	90,120	Long-stay and Short-stay	Cross-sectional		x					
Bardenheier [25]	1174	13,507	Long-stay and Short-stay	Cross-sectional							x
Bardenheier [26]	14,998	235,932	Long-stay and Short-stay	Cross-sectional	x						
Bardenheier [27]	17,043	6,614,939	Short-stay	Cross-sectional	x		x				
Bardenheier [28]	14,237	2,233,392	Long-stay and Short-stay	Cross-sectional	x						
Barrett [29]	35	2570	Long-stay and Short-stay	Longitudinal							x
Bates-Jensen [30]	19	142	Long-stay and Short-stay	Longitudinal							x
Bliss [31]	444	28,119	Long-stay and Short-stay	Longitudinal	x	x					x
Bliss [35]	444	28,119	Long-stay and Short-stay	Longitudinal	x	x					x
Bliss [34]	446	42,693	Long-stay and Short-stay	Longitudinal	x	x					
Bliss [38]		90,580	Long-stay	Longitudinal	x	x					x
Bliss [32]	448	10,713	Long-stay	Longitudinal	x						x
Bliss [33]	451	42,693	Long-stay and Short-stay	Cross-sectional	x	x					x
Bliss [36]	439	10,861	Long-stay	Longitudinal	x	x					x
Bliss [37]		15,927	Long-stay	Longitudinal	x	x					x
Bowblis [39]	355	11,126	Long-stay	Cross-sectional	x	x					x
Brennan [103]		13,507	Long-stay and Short-stay	Cross-sectional							x
Brennan [40]		8300	Long-stay and Short-stay	Cross-sectional	x						
Cai [42]	619	59,740	Long-stay	Cross-sectional	x	x					
Cai [41]	14,332	857,740	Long-stay	Longitudinal	x	x					
Cai [44]		394,948	Long-stay	Cross-sectional	x		x				
Cai [43]		7,734,348	Long-stay	Longitudinal	x		x				
Campbell [93]	14,000		Long-stay and Short-stay	Longitudinal		x		x			
Cassie [45]	1174	13,507	Long-stay and Short-stay	Cross-sectional							x
Chang [46]	511		Long-stay and Short-stay	Cross-sectional							x
Davila [104]	364	9852	Long-stay	Mixed methods	x						
Dufour [47]	358		Long-stay and Short-stay	Cross-sectional	x						x
Engeda [102]	971		Short-stay	Cross-sectional				x	x		x
Estrada [48]	869	55,682	Long-stay	Cross-sectional	x	x					x
Fashaw [49]	12,964		Long-stay and Short-stay	Longitudinal	x	x					x
Fashaw-Walters [50]	15,804	1,200,000	Long-stay	Longitudinal	x						
Frahm [51]		183,841	Long-stay	Cross-sectional	x						
Frahm [52]		88,416	Long-stay	Cross-sectional	x						
Gerardo [94]		74,343	Long-stay	Cross-sectional							
Goel [95]	11,469		Long-stay and Short-stay	Cross-sectional				x			x
Gorges [53]	13,312		Long-stay and Short-stay	Cross-sectional				x	x		
Gruneir [54]	8997	516,082	Long-stay	Cross-sectional	x	x					
Hefele [55]	15,000	3,000,000	Long-stay	Cross-sectional	x			x			
Jesdale [56]	12,096	383,757	Long-stay and Short-stay	Cross-sectional	x						x
Kang [57]	1174	12,507	Long-stay and Short-stay	Cross-sectional							x
Lepore [58]	8732	288,202	Long-stay and Short-stay	Cross-sectional	x	x	x			x	
Li [67]		12,832	Long-stay and Short-stay	Cross-sectional							x
Li [64]	12,473	2,446,808	Long-stay	Longitudinal	x	x				x	
Li [68]		960,644	Short-stay	Cross-sectional	x	x				x	
Li [59]	15,204	868,011	Long-stay	Cross-sectional	x	x				x	
Li [60]	85,941		Long-stay and Short-stay	Longitudinal	x	x	x	x			
Li [65]	15,041	1,302,511	Short-stay	Cross-sectional	x		x				
Li [66]	12,000–13,500 facilities for each year		Long-stay and Short-stay	Longitudinal		x		x			x
Li [62]		1,614,096	Long-stay	Cross-sectional	x	x					
Li [63]	12,576		Long-stay and Short-stay	Cross-sectional	x		x	x	x	x	x
Li [61]	211		Long-stay and Short-stay	Longitudinal				x	x	x	x
Mack [96]		342,920	Long-stay and Short-stay	Cross-sectional	x						
Mathuba [69]	14,312	428,788	Short-stay	Cross-sectional	x		x				
Meyers [97]	4178		Long-stay and Short-stay	Cross-sectional	x	x		x	x		x
Morrison [70]		994,510	Long-stay and Short-stay	Cross-sectional	x						
Nalls [71]	12	336	Long-stay and Short-stay	Cross-sectional							x
Nee [72]		56,194	Long-stay and Short-stay	Longitudinal							
Pu [73]	15,639		Short-stay	Cross-sectional					x		x
Resnick [74]	55	553	Long-stay and Short-stay	Cross-sectional							x
Reynolds [75]	12	1133	Long-stay and Short-stay	Cross-sectional	x						x
Reynolds [76]	6	551	Long-stay and Short-stay	Cross-sectional	x						x
Riester [77]		1,659,966	Long-stay and Short-stay	Longitudinal	x	x	x	x			
Rivera-Hernandez [80]	8375	649,187	Short-stay	Cross-sectional	x	x	x	x			
Rivera-Hernandez [81]	13,375	1,813,963	Short-stay	Cross-sectional	x		x	x	x		x
Rivera-Hernandez [78]	8884	177,396	Short-stay	Cross-sectional	x	x	x				
Rivera-Hernandez [79]	15,137	100,578	Long-stay	Cross-sectional	x	x	x	x			
Rosen [82]	1		Long-stay and Short-stay	Experimental (non-randomized trial)							x
Sengupta [83]		6332	Long-stay and Short-stay	Cross-sectional							x
Shippee [84]	376	10,929	Long-stay and Short-stay	Cross-sectional	x						x
Shippee [85]	376	60,093	Long-stay	Longitudinal	x	x					x
Shippee [86]	355	11,126	Long-stay and Short-stay	Cross-sectional	x						x
Shippee [105]	6	96	Long-stay	Mixed methods	x						
Siegel [87]		124,431	Long-stay and Short-stay	Cross-sectional	x	x					
Strully [101]	1140	12,501	Long-stay	Cross-sectional							x
Temkin-Greener [89]	13,206	915,688	Long-stay	Cross-sectional	x		x				
Temkin-Greener [88]	14,595	65,033	Long-stay	Cross-sectional	x		x		x		
Travers [91]	742	107,874	Long-stay and Short-stay	Cross-sectional	x	x					
Travers [90]	11,587		Long-stay	Cross-sectional				x	x		x
Xu [98]	13,260		Long-stay	Cross-sectional	x	x	x				x
Zheng [92]	555	49,048	Long-stay and Short-stay	Cross-sectional	x		x				
Zisselman [99]	2	290	Long-stay and Short-stay	Cross-sectional	x						
Zuo [100]		597,966	Long-stay and Short-stay	Cross-sectional	x						

AHRF: Area Health Resource Files, BIPOC: Black, Indigenous, and People of Color, CASPER: Certification and Survey Provider Enhanced Reporting, MDS: Minimum Data Set, NH: Nursing home, OSCAR: The Online Survey Certification and Reporting, SNF: Skilled nursing facility, ‘.’: Not stated

^a^ Long-stay is differently defined in the different studies, some state at least 30, some 90, days in NH

### Study design

Of the 82 included studies, 80 (98%) [24–103] were quantitative studies, and two (2%) were mixed methods [104, 105]. The majority of the quantitative studies had an observational cross-sectional design (n = 60, 73%), while 21 (26%) relied upon a longitudinal design [29–35, 37, 38, 41, 43, 49, 50, 61, 65, 66, 68, 72, 77, 86, 93], and one (1%) used a randomized trial design [82] (Table 1).

### Data sources

In terms of data sources, the majority of included studies (*n* = 62, 76%) [25–28, 31–44, 47–52, 54–56, 58–60, 62–66, 68–70, 75–81, 84–89, 91–94, 96–100, 104, 105] used administrative data to study health disparities among nursing home residents. The most prevalent data source used was the Minimum Data Set (MDS) (*n* = 60, 73%) [25–28, 31–44, 47–52, 54–56, 58–60, 62–64, 66, 68–70, 75–81, 84–89, 91, 92, 94, 96–100, 104, 105], a required comprehensive clinical assessment tool conducted on all residents in Medicare and Medicaid-certified nursing homes on admission and periodically thereafter. The MDS includes variables such as demographic information, health conditions, measures of physical and cognitive functioning, mood assessment, and resident preferences.

Thirty-two studies (39%) [24, 31–36, 38, 39, 41, 42, 48, 49, 54, 58, 59, 62, 64–66, 68, 77–79, 81, 86, 87, 91, 93, 94, 97, 98] used Certification and Survey Provider Enhanced Reporting (CASPER) or Online Survey Certification and Reporting System (OSCAR) (i.e. the predecessor to CASPER) data, containing facility-level characteristics. These data are collected during annual inspections and include facility size, staffing levels, and ownership type.

Medicare claims data were used in 17 (21%) studies [28, 43, 44, 58, 60, 63, 66, 69, 77–81, 88, 89, 92, 98]. The most commonly used Medicare files were the Master Beneficiary Summary File, which contains information on Medicare and Medicaid eligibility, enrollment, demographics, chronic conditions, and vital statistics; and the Medicare Provider and Analysis Review file, which includes claims for inpatient hospital and skilled nursing facility stays that were covered by Medicare. These datasets were usually linked with other administrative data sources such as MDS and CASPER (Table 1). It is worth noting that, in the United States, Medicare is the primary health insurance for adults age 65 + as well as a subset of disabled and chronically ill younger adults. As such, it effectively covers the entire nursing home population and thus is an apt data source to measure health services and outcomes for this population.

Other data sources were commonly used to measure facility- and area-level characteristics. This included LTCFocus (n = 15, 18%) [53, 55, 61, 63, 65, 66, 77, 79–81, 90, 93, 95, 97, 102], which includes aggregate nursing home-level measures derived from several sources, including MDS and CASPER. LTCFocus measures include structural facility characteristics, characteristics of the resident populations, market characteristics, and state policies relevant to long-term care services and financing (Brown University School of Public Health). Ten (12%) studies [30, 53, 61, 63, 73, 80, 88, 90, 97, 102] used CMS Care Compare data, which include quality ratings, quality measures, and key facility characteristics (Centers for Medicare & Medicaid Services). Six (7%) [58, 59, 61, 63, 64, 68] used the Area Health Resources file (Health Resources & Services Administration), which provides information on population characteristics, economics, and environment at the county, state, and national level.

The remaining data sources were classified as ‘Other’ and included a large variety of sources. Some examples of ‘other’ data sources were medical records, surveys, and patient-reported.

outcome measures, state and local public health data, and U.S. census data (Table 1).

### Independent variable(s) and covariates

Health disparities among nursing home residents may be measured by different characteristics (Table 2). The majority of studies (*n* = 75, 91%) focused on a single characteristic (e.g., race), while six studies (7%) [47, 49, 72, 76, 87, 99] examined the intersection of multiple characteristics (e.g., race and socioeconomic factors). The characteristics examined among reviewed studies included: race and ethnicity (*n* = 71, 87%) [24–27, 29–39, 41–56, 58–72, 74, 76–88, 90–94, 96, 99–102, 105], clinical conditions (*n* = 7, 9%) [28, 40, 47, 75, 95, 97, 103], rural–urban location of the nursing home (*n* = 4, 5%) [57, 73, 89, 98, 102], socioeconomic factors (*n* = 4, 5%) [49, 72, 87, 99], sex (*n* = 1, 1%) [104], and age (*n* = 1, 1%) [76].

**Table 2:** Primary independent variable(s), covariates, and outcomes

First author, publication year	Primary independent variable(s)	Operationalization of primary independent variable(s)	Measurement level of primary independent variable(s)	Primary outcome(s) measurement(s)	Primary outcome(s) category	Measurement level of primary outcome variable(s)	Controls	Within and/or across nursing homes differences
Bardenheier [24]	Race and/or ethnicity	Black and White. Percentage of Black residents in NH	Resident level Facility level	Percentage vaccinated, refused or not offered Vaccination rates	Vaccination (flu and/or pneumococcal)	Resident level Facility level	Individual resident demographic characteristics Facility structural characteristics Facility processual factors	Within and across
Bardenheier [25]	Race and/or ethnicity	Black and White	Resident level	Percentage vaccinated, refused or not offered Vaccination rates	Vaccination (flu and/or pneumococcal)	Resident level	Individual resident demographic characteristics Individual resident clinical characteristics Facility structural characteristics Facility processual factors State or larger geographical area characteristics	Across
Bardenheier [26]	Race and/or ethnicity	Black and White Percentage of Black residents in NH	Resident level Facility level	Percentage vaccinated, refused or not offered Vaccination rates	Vaccination (flu and/or pneumococcal)	Resident level Facility level	Individual resident demographic characteristics Facility structural characteristics	Within and across
Bardenheier [27]	Clinical condition	With and without Alzheimer’s disease and related Dementias	Resident level	Percentage discharged from SNF, who became long-stay residents in SNF	(re-)hospitalization, emergency room use Successful discharge to community	Resident level	Individual resident demographic characteristics Community, county or zip code characteristics	Across
Bardenheier [28]	Race and/or ethnicity	Black and White Percentage of Black residents in NH	Resident level Facility level	Percentage vaccinated, refused or not offered Vaccination rates	Vaccination (flu and/or pneumococcal)	Resident level Facility level	Individual resident demographic characteristics Facility structural characteristics State or larger geographical area characteristics	Across
Barrett [29]	Race and/or ethnicity	Black and White Ratio of Black to White residents in facility	Resident level Facility level	Percentage vaccinated	Vaccination (flu and/or pneumococcal)	Facility level	Facility structural characteristics Facility processual factors	Within and across
Bates-Jensen [30]	Race and/or ethnicity	Black, Asian, Hispanic, White	Resident level	Percentage with pressure injuries, stage 4 pressure injuries	Pressure ulcer, wound	Resident level		Across
Bliss [31]	Race and/or ethnicity	AIAN, API, Black, White, and Hispanic	Resident level	Proportion with incontinence, whose incontinence is cured Duration until incontinence is cured	(in)continence (management)	Resident level	Individual resident demographic characteristics Individual resident clinical characteristics Facility structural characteristics Facility processual factors Community, county or zip code characteristics	Across
Bliss [35]	Race and/or ethnicity	AIAN, API, Black, White, and Hispanic	Resident level	Proportion with incontinence, whose incontinence is cured Duration until incontinence is cured	(in)continence (management)	Resident level	Individual resident demographic characteristics Individual resident clinical characteristics Facility structural characteristics	Across
Bliss [34]	Race and/or ethnicity	AIAN, API, Black, White, and Hispanic	Resident level	Proportion deveolping incontinence Duration until developed incontinence	(in)continence (management)	Resident level	Individual resident demographic characteristics Individual resident clinical characteristics Facility structural characteristics Community, county or zip code characteristics	Across
Bliss [38]	Race and/or ethnicity	AIAN, API, Black, White, and Hispanic	Resident level	Proportion developing pressure ulcers Duration until developed pressure ulcers Proportion receiving pressure ulcer treatments	Pressure ulcer, wound	Resident level	Individual resident clinical characteristics Facility structural characteristics Facility processual factors Community, county or zip code characteristics	Across
Bliss [32]	Race and/or ethnicity	AIAN, API, Black, White, and Hispanic	Resident level	Proportion receiving prevention for incontinence-associated skin damage	Pressure ulcer, wound	Resident level	Individual resident clinical characteristics Facility processual factors	Across
Bliss [33]	Race and/or ethnicity	AIAN, API, Black, White, and Hispanic	Resident level	Proportion receiving incontinence prevention	(in)continence (management)	Resident level	Individual resident demographic characteristics Individual resident clinical characteristics Facility structural characteristics Facility processual factors	Across
Bliss [36]	Race and/or ethnicity	AIAN, API, Black, White, and Hispanic	Resident level	Proportion whose pressure ulcers healed	Pressure ulcer, wound	Resident level	Individual resident demographic characteristics Individual resident clinical characteristics Facility structural characteristics	Across
Bliss [37]	Race and/or ethnicity	AIAN, API, Black, White, and Hispanic	Resident level	Proportions with low social engagement	Social engagement	Resident level	Individual resident clinical characteristics Facility structural characteristics Community, county or zip code characteristics	Across
Bowblis [39]	Race and/or ethnicity	BIPOC and White High/low BIPOC composition of NH	Resident level Facility level	Mean overall quality of life score and domain scores	Quality of life	Resident level	Individual resident demographic characteristics Individual resident clinical characteristics Facility structural characteristics Facility processual factors	Across
Brennan [103]	Clinical condition	With and without Dementia, Depression, Serious Mental Illness, Substance Abuse Disorder, Dual Diagnosis	Resident level	Proportion with pain, lower or"missing/don't know"pain severity ratings, obtaining opioids, non-opioid pain medications only	Pain (management)	Resident level	Individual resident demographic characteristics Individual resident clinical characteristics	Across
Brennan [40]	Clinical condition	With and without Dementia and serious mental illness	Resident level	Proportion with pain, obtaining no treatment, receiving as-needed pain medication	Pain (management)	Resident level	Individual resident demographic characteristics Individual resident clinical characteristics	Across
Cai [42]	Race and/or ethnicity	Black and White. Proportion of Black residents	Resident level Facility level	Percentage with pressure ulcers Pressure ulcers rates	Pressure ulcer, wound	Resident level	Individual resident demographic characteristics Individual resident clinical Facility structural characteristics Facility processual factors	Within and across
Cai [41]	Race and/or ethnicity	Black and White. Proportion of Blacks in facility	Resident level Facility level	Percentage vaccinated Vaccination rates	Vaccination (flu and/or pneumococcal)	Facility level	Individual resident demographic characteristics Facility structural characteristics	Within and across
Cai [44]	Race and/or ethnicity	Black and White	Resident level	Percentage hospitalized at end of life Proportion with(out) DNH orders	(re-)hospitalization, emergency room use Palliative or End-of-life care	Resident level	Individual resident demographic characteristics Individual resident clinical characteristics	Within
Cai [43]	Race and/or ethnicity	Black and White. Percentage of Black residents in facility	Resident level Facility level	Percentage diagnosed with schizophrenia Rate of schizophrenia diagnoses	Diagnosis of psychiatric condition	Resident level	Individual resident demographic characteristics Facility structural characteristics Facility processual factors	Within and across
Campbell [93]	Race and/or ethnicity	NH concentration of racial/ethnic minority resident	Facility level	Average number of deficiencies	Quality of life	Facility level	Facility structural characteristics Facility processual factors	Across
Cassie [45]	Race and/or ethnicity	Black and White	Resident level	Percentage of specific types of restraints Proportion of any type of restraint	Physical restraint use	Resident level	Individual resident demographic characteristics Individual resident clinical characteristics	Across
Chang [46]	Race and/or ethnicity	Percentage of Black residents in facility	Facility level	Percentage of high-risk residents who have pressure ulcers Percentage who were physically restrained Percentage of eligible residents vaccinated	Vaccination (flu and/or pneumococcal) Pressure ulcer, wound Physical restraint use	Facility level	Facility structural characteristics	Across
Davila [104]	Gender or sex	Male and Female	Resident level	Mean quality of life scores	Quality of life	Resident level	Individual resident demographic characteristics Individual resident clinical characteristics Facility structural characteristics Facility processual factors	Across
Dufour [47]	Race and/or ethnicity Clinical condition	Percent of residents in facility who were non-White or had Dementia	Facility level	Average weekly rates of new Covid-19 infections	Covid-19 cases and/or deaths	Facility level	Individual resident demographic characteristics Individual resident clinical characteristics Facility structural characteristics Community, county or zip code characteristics	Across
Engeda [102]	Race and/or ethnicity	Majority White residents; High proportion of Black or Hispanic/Latinx residents; High proportions of Asian residents; Or racially and ethnically mixed	Facility level	Covid-19 incidence rates	Covid-19 cases and/or deaths	Facility level	Individual resident demographic characteristics Facility structural characteristics Facility processual factors Community, county or zip code characteristics	Across
Estrada [48]	Race and/or ethnicity	Concentration of Black and Hispanic residents in NH	Facility level	Mean scores of palliative care services (higher scores indicated more services)	Palliative or End-of-life care	Facility level	Facility structural characteristics	Across
Fashaw [49]	Race and/or ethnicity; Social and/or economic factors	High proportions of Black residents The percentage of residents with Medicaid as their primary payer	Facility level	Rates of inappropriate antipsychotic	Antipsychotic use	Facility level	Individual resident demographic characteristics Individual resident clinical characteristics Facility structural characteristics Facility processual factors	Across
Fashaw-Walters [50]	Race and/or ethnicity	Black and non-Black	Resident level	Prevalence rates of schizophrenia	Diagnosis of psychiatric condition	Resident level	Individual resident demographic characteristics Individual resident clinical characteristics	Within
Frahm [51]	Race and/or ethnicity	Black, White, Hispanic, and Asian	Resident level	Proportions of residents with documented advanced directives, hospice use, and hospitalizations	Hospice use (re-)hospitalization, emergency room use Advanced care planning	Resident level	Individual resident demographic characteristics Individual resident clinical characteristics	
Frahm [52]	Race and/or ethnicity	Black, White, Hispanic, and Asian	Resident level	Proportions of residents with documented advanced directives, hospice use, hospitalizations, who died	(re-)hospitalization, emergency room use Advanced care planning Death	Resident level	Individual resident demographic characteristics Individual resident clinical characteristics	Across
Gerardo [94]	Race and/or ethnicity	Hispanics, non-Hispanic, non-Hispanic Whites Nursing Home Concentration of Hispanics	Resident level Facility level	Percentage with stage 2–4 pressure ulcers	Pressure ulcer, wound	Resident level	Individual resident demographic characteristics Individual resident clinical characteristics Facility structural characteristics	Across
Goel [95]	Clinical condition	The proportion of residents with low cognitive functioning	Facility level	Average number of RN hours per resident day, staffing rating and total nurse staffing rating	Staffing	Facility level	Facility structural characteristics Facility processual factors Community, county or zip code characteristics	Across
Gorges [53]	Race and/or ethnicity	Concentration of Black and Hispanic residents in NH	Facility level	Mean number of deaths	Covid-19 cases and/or deaths	Facility level	Facility structural characteristics Facility processual factors Community, county or zip code characteristics	Across
Gruneir [54]	Race and/or ethnicity	Black, White, and Other Percentage of Black residents	Resident level Facility level	Percentages of residents hospitalized and odds of hospitalization	(re-)hospitalization, emergency room use	Resident level	Individual resident demographic characteristics Individual resident clinical characteristics Facility structural characteristics State or larger geographical area characteristics	Across
Hefele [55]	Race and/or ethnicity	Black, White and Hispanics	Resident level	Percentage at low risk for incontinence, who have lost too much weight, with urinary tract infections, with pressure ulcers, with worsening depression or anxiety, decline in late-loss activities of daily living	Depression (management) Pressure ulcer, wound Physical restraint use Weight loss ADL (in)continence (management) Urinary tract infection	Resident level		Within
Jesdale [56]	Race and/or ethnicity	Black, White and Hispanics White, mostly Black, mostly Hispanic, or integrate NH	Resident level Facility level	Percentage with pain	Pain (management)	Resident level	Individual resident demographic characteristics Individual resident clinical characteristics	Within and across
Kang [57]	Urban–rural	Metropolitan, micropolitan, and rural areas	Facility level	Odds ratio of being hospitalized, receiving vaccine, experiencing moderate to severe pain	(re-)hospitalization, emergency room use Vaccination (flu and/or pneumococcal) Pain (management)		Individual resident demographic characteristics Individual resident clinical characteristics Facility structural characteristics Facility processual factors	Across
Lepore [58]	Race and/or ethnicity	Black and White	Resident level	Odds ratio of using hospice Percentage using hospice	Hospice use	Resident level	Individual resident demographic characteristics Individual resident clinical characteristics Facility structural characteristics Facility processual factors Community, county or zip code characteristics	Across
Li [67]	Race and/or ethnicity	Black and White	Resident level	Percentage vaccinated Vaccination rates Odds ratio of being unvaccinated	Vaccination (flu and/or pneumococcal)	Resident level	Individual resident demographic characteristics Individual resident clinical characteristics Facility structural characteristics	Across
Li [64]	Race and/or ethnicity	Black and White Proportion of residents who were Black	Resident level Facility level	Percentage with pressure ulcers Odds ratio of having pressure ulcers	Pressure ulcer, wound	Resident level	Individual resident demographic characteristics Individual resident clinical characteristics Facility structural characteristics Facility processual factors Community, county or zip code characteristics	Across
Li [68]	Race and/or ethnicity	Black and White	Resident level	30-and 90-day rehospitalization rate	(re-)hospitalization, emergency room use	Resident level	Individual resident demographic characteristics Individual resident clinical characteristics Facility structural characteristics Community, county or zip code characteristics	Across
Li [59]	Race and/or ethnicity	Black, White, and Hispanic	Resident level	Mean social engagement score	Social engagement	Resident level	Individual resident demographic characteristics Individual resident clinical characteristics Facility structural characteristics Community, county or zip code characteristics	Across
Li [60]	Race and/or ethnicity	Percentage of racial and/or ethnic minority residents	Facility level	Average annual number of health care-related deficiencies Percentage with serious deficiencies	Facility deficiencies and/or citations	Facility level	Facility structural characteristics Facility processual factors	Across
Li [65]	Race and/or ethnicity	Black and White Proportions of Blacks	Resident level Facility level	30-day all-cause rehospitalization rate 30-day potentially avoidable rehospitalization rate	(re-)hospitalization, emergency room use	Resident level	Facility structural characteristics	Within and across
Li [66]	Race and/or ethnicity	Percentage of racial/ethnic minority residents	Facility level	Mean RN, LPN, CNA hours per resident day	Staffing	Facility level	Facility structural characteristics Community, county or zip code characteristics State or larger geographical area characteristics	Across
Li [62]	Race and/or ethnicity	Black, White, Hispanic, and API	Resident level	Percentage with significant depressive symptoms	Depression (management)	Resident level	Individual resident demographic characteristics Individual resident clinical characteristics Facility structural characteristics Facility processual factors	Within and across
Li [63]	Race and/or ethnicity	Percentage of racial/ethnic minority residents	Facility level	Mean number of weekly new Covid-19 confirmed cases among residents Odds ratio of having at least one new resident case Mean number of weekly new Covid-19 deaths among residents	Covid-19 cases and/or deaths	Facility level	Facility structural characteristics Facility processual factors	Across
Li [61]	Race and/or ethnicity	Percentage of racial/ethnic minority residents	Facility level	Mean number of Covid-19 cases Incidence rate ratio (IRR) for high-proportion group Mean number of weekly Covid-19-related deaths	Covid-19 cases and/or deaths	Facility level	Facility structural characteristics Facility processual factors Community, county or zip code characteristics	Across
Mack [96]	Race and/or ethnicity	Black and White	Resident level	Percentage with pain Proportion using scheduled analgesics, pro re nata (as needed) analgesics, and non-pharmacological methods	Pain (management)	Resident level	Individual resident demographic characteristics Individual resident clinical characteristics	Across
Mathuba [69]	Race and/or ethnicity	Black, White, Hispanic and Other	Resident level	Percentage and odds ratio with any improvement in ADL function	ADL	Resident level	Individual resident demographic characteristics Individual resident clinical characteristics	Across
Meyers [97]	Clinical condition	HIV and non-HIV NH HIV Concentration	Resident level Facility level	Average rating of quality Percentage re-hospitalized Average number of deficiencies Proportion subjected to physical restraints	Facility deficiencies and/or citations Physical restraint use Facility star-rating (re-)hospitalization	Facility level	Facility structural characteristics Community, county or zip code characteristics State or larger geographical area characteristics	Across
Morrison [70]	Race and/or ethnicity	Black, White, and Hispanics	Resident level	Percentage with vocal complaints of pain Adjusted prevalence ratio for vocal complaints of pain Percentage receiving any pharmacologic pain intervention Adjusted prevalence ratio for receiving any pharmacologic pain intervention	Pain (management)	Resident level	Individual resident demographic characteristics Individual resident clinical characteristics	Across
Nalls [71]	Race and/or ethnicity	Black and White	Resident level	Percentage and odds ratio of being treated with antidepressants	Depression (management)	Resident level	Individual resident demographic characteristics Individual resident clinical characteristics	Across
Nee [72]	Race and/or ethnicity; Social and/or economic factors	Black, White, and Hispanics. Dual eligibility status	Resident level	Adjusted hazard ratio for risk of death	Death	Resident level	Individual resident demographic characteristics Individual resident clinical characteristics Community, county or zip code characteristics	Across
Pu [73]	Urban–rural	Urban–rural	Facility level	Percentage with up-to-date vaccinations Vaccination rates	Vaccination (flu and/or pneumococcal)	Facility level	Facility structural characteristics Facility processual factors	Across
Resnick [74]	Race and/or ethnicity	Black and White	Resident level	Percentage decrease in agitation Mean quality of life score Percentage increase in person-centered care approaches Percentage decrease in number of falls Percentage of re-hospitalization Percentage with vocal complaints of pain Percentage treated with antidepressants	Quality of life (re-)hospitalization, emergency room use Pain (management) Depression (management) Falls ADL Behavioral problems (management), mood symptoms Quality-of-care interactions between staff and resident	Resident level	Individual resident demographic characteristics Individual resident clinical characteristics Facility structural characteristics Facility processual factors	Across
Reynolds [75]	Race and/or ethnicity; Age	White and Minority Age: 22–79, 80–87, 88 +	Resident level	Odds ratio of having DNR orders Percentage with living wills Percentage with health care proxies Mean number of pain medications Percentage receiving pain medication Percentage with pain Percentage with documentation of in-depth advanced care plan discussions	Advanced care planning Pain (management)	Resident level	Individual resident demographic characteristics Individual resident clinical characteristics	Across
Reynolds [76]	Clinical condition	No, mild, moderate, and severe cognitive impairment	Resident level	Percentage with pain Percentage receiving pain medications	Pain (management)	Resident level	Individual resident demographic characteristics Individual resident clinical characteristics	Across
Riester [77]	Race and/or ethnicity	Black, White, Hispanic	Resident level	Percentage vaccinated	Vaccination (flu and/or pneumococcal)	Resident level	Individual resident demographic characteristics Facility structural characteristics Facility processual factors	Across
Rivera-Hernandez [80]	Race and/or ethnicity	Percentage of Blacks and Hispanics in SNF	Facility level	Percentage successfully discharged to the community 30-day rehospitalization rate	(re-)hospitalization, emergency room use Successful discharge to community NH star-rating	Facility level	Facility structural characteristics Facility processual factors	Across
Rivera-Hernandez [1]	Race and/or ethnicity	Black, White, and Hispanic	Resident level	Readmission Rate	(re-)hospitalization, emergency room use	Resident level	Individual resident demographic characteristics Individual resident clinical characteristics Facility structural characteristics Facility processual factors	Within and across
Rivera-Hernandez [78]	Race and/or ethnicity	Proportion of Hispanic residents	Facility level	Percentage successfully discharged to the community 30-day rehospitalization rate	(re-)hospitalization, emergency room use Successful discharge to community NH star-rating	Facility level	Facility structural characteristics Facility processual factors Community, county or zip code characteristics	Across
Rivera-Hernandez [79]	Race and/or ethnicity	Black, White, Hispanic, API, AIAN	Resident level	Percentage vaccinated, received antipsychotic medication, experienced one or more falls with major injury, were physically restrained, lost too much weight	Vaccination (flu and/or pneumococcal) Falls Physical restraint use Weight loss Antipsychotic use	Resident level	Individual resident demographic characteristics Individual resident clinical characteristics Facility structural characteristics Facility processual factors	Within and across
Rosen [82]	Race and/or ethnicity	Black and White	Resident level	Percentage who developed stage 1–4 pressure ulcers	Pressure ulcer, wound	Resident level		Across
Sengupta [83]	Race and/or ethnicity	White and Non-White	Resident level	Percentage with dementia receiving special dementia care	Specialized Dementia care	Resident level	Individual resident demographic characteristics Facility structural characteristics	Across
Shippee [84]	Race and/or ethnicity	White and Non-White Percentage of White residents in NH	Resident level Facility level	Mean scores of various quality of life domains	Quality of life	Resident level Facility level	Individual resident demographic characteristics Individual resident clinical characteristics Facility structural characteristics Facility processual factors	Across
Shippee [85]	Race and/or ethnicity	White and minority. Proportion of minority residents	Resident level Facility level	Mean quality of life score	Quality of life	Resident level	Individual resident demographic characteristics Individual resident clinical characteristics Facility structural characteristics Facility processual factors	Across
Shippee [86]	Race and/or ethnicity	White and minority Proportion of minority residents	Resident level Facility level	Mean quality of life score	Quality of life	Resident level	Individual resident demographic characteristics Individual resident clinical characteristics Facility structural characteristics Facility processual factors	Across
Shippee [105]	Race and/or ethnicity	Proportion of BIPOC residents	Facility level	Mean quality of life score Experiences with Quality of Life	Quality of life	Resident level	Individual resident demographic characteristics Individual resident clinical characteristics Facility structural characteristics Facility processual factors	Across
Siegel [87]	Race and/or ethnicity Social and/or economic factors	Black and White High school diploma and above, and high school and less	Resident level	Odds ratio of receiving treatment for depression	Depression (management)	Resident level	Individual resident demographic characteristics Individual resident clinical characteristics Facility structural characteristics Facility processual factors	Across
Strully [101]	Race and/or ethnicity	Black, White, and Other Proportion of Black residents	Resident level Facility level	Odds ratio of being vaccinated	Vaccination (flu and/or pneumococcal)	Resident level Facility level	Individual resident demographic characteristics Individual resident clinical characteristics Facility structural characteristics	Across
Temkin-Greener [89]	Urban–rural	Urban–rural	Facility level	Prevalence of death in hospital and hospice use	Place of tere Pain (management) Hospice use	Resident level	Individual resident demographic characteristics Individual resident clinical characteristics Facility structural characteristics Facility processual factors	Across
Temkin-Greener [88]	Race and/or ethnicity	Black and White Proportion of Black residents	Resident level Facility level	Proportion hospitalized within 30 days of death	(re-)hospitalization, emergency room use	Resident level	Individual resident demographic characteristics Individual resident clinical characteristics Facility structural characteristics Facility processual factors	Within and across
Travers [91]	Race and/or ethnicity	Black, White and Hispanics	Resident level	Odds ratio of being vaccinated	Vaccination (flu and/or pneumococcal)	Resident level	Individual resident demographic characteristics Individual resident clinical characteristics Facility structural characteristics Community, county or zip code characteristics	Within and across
Travers [90]	Race and/or ethnicity	Proportion of Black residents	Facility level	Prevalence of Covid-19 infections and deaths	Covid-19 cases and/or deaths	Facility level	Facility structural characteristics Facility processual factors Community, county or zip code characteristics	Across
Xu [98]	Urban–rural	Urban–rural	Facility level	Percentage with any ED visit, outpatient ED visit, potentially avoidable ED visits	(re-)hospitalization, emergency room use	Facility level	Facility structural characteristics Facility processual factors	Across
Zheng [92]	Race and/or ethnicity	Black and White. Proportion of Black residents	Resident level Facility level	Percentage and odds ratio of in-hospital death and hospice use	Place of death Hospice use	Resident level	Individual resident demographic characteristics Individual resident clinical characteristics	Within and across
Zisselman [99]	Race and/or ethnicity; Social and/or economic factors	Black and White Medicaid status	Resident level	Proportion with recorded psychiatric diagnoses, displaying behavior symptoms, receiving psychotropic medications	Behavioral problems (management), mood symptoms Antipsychotic use Diagnosis of psychiatric condition	Resident level	Individual resident demographic characteristics Individual resident clinical characteristics Facility structural characteristics	Across
Zuo [100]	Race and/or ethnicity	Black, White and Other	Resident level	Percentage with indwelling catheters, intermittent catheterization	Urinary catheterization	Resident level	Individual resident demographic characteristics Individual resident clinical characteristics Facility processual factors	Across

ADL: Activities of Daily Living, AIAN: American Indian or Alaskan Native, API: Asian or Pacific Islander, BIPOC: Black, Indigenous, and People of Color, CAN: Certified Nursing Assistant, DNR: Do-Not-Resuscitate, ED: Emergency Department, LPN: Licensed Practical Nurse, RN: Registered Nurse, SNF: Skilled nursing facility

Race and ethnicity were the most studied characteristics. Forty-three (61%) incorporated race and/or ethnicity measures at the resident level, 12 studies (17%) [46–49, 53, 61, 65, 66, 78, 81, 93, 102, 106] included aggregate facility-level measures of a nursing home’s population, while 14 (20%) included both [25–27, 29, 42, 43, 56, 60, 84–86, 92, 94, 101].

Usually, two racial and/or ethnic groups were compared; the most prevalent comparison was between Black and White residents (*n* = 21, 30%) [24–27, 29, 41–45, 58, 60, 67, 68, 71, 74, 82, 87, 88, 96, 99]. In 17 (24%) studies [39, 47, 50, 54, 59, 69, 72, 76–78, 83, 85, 86, 100–102, 105], certain race or ethnicity groups were aggregated into categories such as “other”, “racial/ethnic minority resident”, “minority”, “Black, Indigenous, People of Color”, “non-Whites”, or “non-Blacks”. This terminology is directly taken from the primary studies included in the review. In many cases, this was done due to small samples of specific racial and/or ethnic groups. Thirty-nine (55%) studies [30–38, 48, 51–56, 59, 61–63, 65, 66, 69, 70, 76–81, 83–86, 91, 94, 100, 102, 105] explicitly reported including Hispanic residents in their analyses, while 24 (34%) [30–38, 51, 52, 61–63, 65, 66, 79, 83–86, 100, 102, 105] included Asian or Pacific Islander (API) and 20 (28%) [31–38, 61, 63, 65, 66, 79, 83–86, 100, 105] included American Indian or Alaskan Native (AIAN). Across all of the studies, very few residents identified as AIAN or API in the studies’ sample (usually less than 1%).

Seven studies (9%) [28, 40, 47, 75, 95, 97, 103] examined clinical conditions as a characteristic, with four of those incorporating person-level measures [28, 40, 75, 103], and three [47, 95, 97] incorporating aggregate facility-level measures of condition or disease prevalence. Selected conditions included dementia and cognitive impairment (*n* = 6) [28, 40, 47, 75, 95, 103], psychiatric conditions (*n* = 2) [40, 103], and Human Immunodeficiency Virus (*n* = 1) [97]. Four studies [57, 73, 89, 98] (5%) examined rural–urban disparities based on the location of the nursing home. Four studies [49, 72, 87, 99] (5%) examined disparities based on socioeconomic factors including facility-level measures of the proportion of residents with Medicaid as primary payer (n = 1) [49]; and person-level measures of dual-Medicare and Medicaid enrollment (n = 2) [72, 99] and education level (*n* = 1) [87]. Finally, only one study (1%) examined disparities by sex, and one (1%) examined disparities by age.

Common person-level covariates used in studies included demographic characteristics (e.g. sex, age, level of education) (*n* = 58, 71%) [24–28, 31, 33, 35, 36, 38–45, 47, 49–52, 54, 56–59, 62, 64, 67–72, 74–77, 79, 80, 83–89, 91, 92, 94, 96, 100–105] and clinical characteristics (e.g. diagnosis, nutritional status, cognitive functioning) (n = 53, 65%) [24, 31–40, 42, 44, 45, 47, 49–52, 54, 56–59, 62, 64, 67–69, 71, 72, 74–76, 79, 80, 84–89, 91, 92, 94, 96, 100, 101, 103–105]. For facility-level covariates, 59 studies (72%) [24–27, 29, 31–36, 38, 39, 41–43, 46–49, 53, 54, 57–68, 73, 74, 77–81, 83–91, 93–95, 97, 98, 101, 102, 104, 105] included structural factors (e.g. nursing home size, staffing levels), and 38 (46%) [24, 25, 29, 31, 34, 36, 37, 39, 42, 43, 49, 53, 58, 61–63, 66, 68, 73, 74, 77–81, 84–90, 93, 95, 98, 100, 104, 105] included processual measures (e.g. presence of protocols/guidelines). Twenty studies (34%) [28, 31, 32, 34, 38, 47, 53, 58, 59, 61, 64, 65, 68, 72, 78, 90, 91, 95, 97, 102] included community, county, or zip code variables as covariates and 4 (5%) [27, 54, 65, 97] included state or larger geographical area characteristics. (e.g., concentration of Medicare Advantage enrollees, dual-eligibles in the population) (Table 2).

We assessed whether the studies examined within and/or across nursing home differences by reviewing their research questions and statistical approach. To be categorized as examining'within nursing home differences,'studies needed to include facility-level fixed or random effects in their models, such that residents were being compared within the same facility. Studies that included facility-level controls with no facility-level fixed or random effect, and studies that included no facility-level controls were classified as examining ‘across nursing home differences’. Studies that included a hybrid of analyses were classified as examining both. Of the 82 studies included in this review, four (5%) [44, 50, 55, 80] primarily focused on within-nursing home differences, 65 (79%) [24, 27, 28, 30–40, 45–49, 51–54, 57–59, 61, 63–78, 81–87, 89, 90, 93–105] on across-nursing home differences, and 13 (16%) [25, 26, 29, 41–43, 56, 60, 62, 79, 88, 91, 92] on both.

### Outcomes

**Fig. 2:**
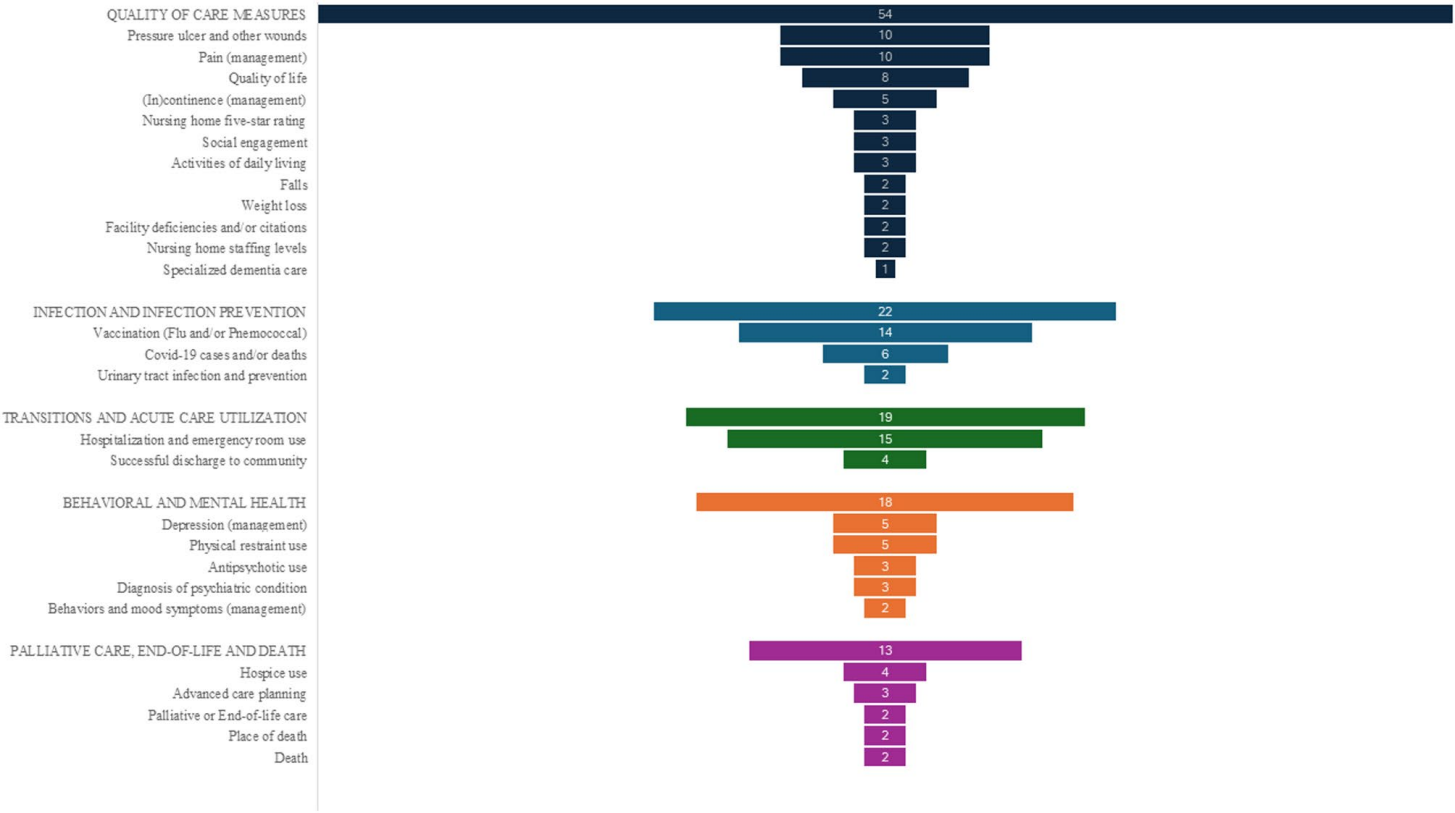
Outcome categories and domains

Outcomes examined by the 82 included studies fell into 27 different categories. These categories were grouped into five domains: 1) Quality of care measures, 2) Infection and infection prevention, 3) Transitions and acute care utilization, 4) Behavioral and mental health, and 5) Palliative care, end-of-life and death (Fig. 2).

Some studies fall into multiple domains as they investigate several outcomes. For example, a study that examines both vaccination and death will fall under the domains of Infection and infection prevention and Palliative care, end-of-life and death. Consequently, when reporting the number of studies per domain, some studies are counted more than once. Furthermore, some studies include multiple categories within the same domain. For instance, a study that investigates both pressure ulcers and pain will be counted under two categories within the domain Quality of care measures. As a result, the total number of categories may exceed the number of included studies.

The largest domain was ‘Quality of care measures’ (*n* = 54,) [30–40, 42, 46, 55–57, 59, 65, 66, 68–70, 74–76, 78, 79, 81–86, 89, 93–97, 103–105], with Pressure ulcer and other wounds (*n* = 10) [30, 33, 34, 37, 42, 46, 55, 68, 82, 94], Pain (management) (*n* = 10) [40, 56, 57, 70, 74–76, 89, 96, 103] and Quality of life (*n* = 8) [39, 74, 84–86, 93, 104, 105] as the most prevalent categories. For the domain ‘Infection and infection prevention’ (*n* = 22) [24–27, 29, 41, 46, 47, 53, 57, 61, 63, 67, 73, 77, 79, 90, 91, 98, 100–102], Vaccination (flu and/or pneumococcal) was the most studied category (*n* = 14) [24–27, 29, 41, 46, 57, 67, 73, 77, 79, 91, 101]. For the domain ‘Transitions and acute care utilization’(*n* = 19) [28, 44, 51, 52, 54, 57, 60, 64, 74, 78–81, 88, 97, 98] had Hospitalization and emergency room use as the most prevalent category (*n* = 15) [28, 44, 51, 52, 54, 57, 60, 64, 74, 78, 80, 81, 88, 97, 98]. For ‘Behavioral and mental health’ (*n* = 18) [43, 45, 46, 49, 50, 55, 58, 62, 71, 74, 79, 87, 97, 99], Depression (management) (*n* = 5) [55, 62, 71, 74, 87] and Physical restraint use (*n* = 5) [45, 46, 55, 79, 97] were the most studied categories. For ‘Palliative and end-of-life’ (*n* = 13) [44, 48, 51, 52, 58, 72, 76, 89, 92], Hospice use was the most studied category (*n* = 4) [51, 58, 89, 92] Across the five domains, the five most prevalent outcome categories studied were Hospitalization and emergency room use (*n* = 15), Vaccination (flu and/or pneumococcal) (*n* = 14), Pressure ulcer and other wounds (*n* = 10), Pain (management) (*n* = 10), and Quality of Life (*n* = 8). The number of outcomes studied per study, ranged from one to nine, using individual person-level outcomes (*n* = 54, 66%), facility-level outcomes (*n* = 21, 26%) [29, 41, 46, 47, 49, 53, 57, 61, 63, 65, 66, 73, 78, 81, 93, 95, 97–100, 102, 105], or both (*n* = 7, 9%) [25–27, 30, 39, 84, 101].

## Discussion

In the 82 studies that met inclusion criteria for review, we noted an apparent influx in published research on nursing home health disparities over the past 10–15 years. The studies ranged from studying health disparities in small, geographically restricted samples of nursing home residents and/or nursing homes to using large, nationally representative samples. Most appeared to include long-stay residents, while the number of studies focusing specifically on short-term residents was limited. The studies focused primarily on racial and/or ethnic disparities, mostly limited to including and comparing Black and White nursing home residents. Studies investigating other factors that can contribute to health disparities in nursing homes, such as the residents’ clinical conditions, sex, gender, age, sexual orientation, religion, disability, and more, were very limited or lacking. A wide range of outcomes were studied, mostly related to the domain ‘Quality of care measures’. The most prevalent single outcome categories were ‘Hospitalization and emergency room use’, ‘Vaccination (flu and/or pneumococcal)’, ‘Pressure ulcers and other wounds’, ‘Pain (management)’, and ‘Quality of Life’. All studies used quantitative methods, with only two incorporating mixed methods, and most relied on administrative data sources. Most were observational cross-sectional studies, and most examined differences in health outcomes across nursing homes, rather than within the same facilities.

Our review highlights a few overarching issues we believe future work could take into consideration. First, the reviewed literature on health disparities in nursing homes largely provides snapshots of health disparities at a single point in time or for a series of cross-sectional comparisons of different cohorts, seeking to establish relationships between variables. There is a need to move beyond description (‘health disparities exist’) towards explanation and examine the underlying structures, processes, and policies contributing to the health disparities in nursing homes. Additional insight is needed to determine how and why health disparities arise, are sustained over time, and what factors exacerbate or protect against poorer health and quality of care outcomes in certain groups in nursing homes. This is where the concept of ‘health inequity’ comes into play.

Although standard definitions of ‘health disparity’ and health inequity’ exist, they are inconsistently applied in the literature. Therefore, our second issue is the need for future research to clearly define their concepts to avoid confusion about the meaning and application of their research. Most papers demonstrate a disparity and call it an inequity without transparently and explicitly discussing how and why they made the linkage. Such arguments are key to advancing our understanding of the differences we observe in health and/or quality of care among nursing home residents, for the creation of successful health policies and avoiding misdirection of attention and resources. More empirical research on how people compare and interpret disparities (e.g., is the difference large or small?) and research into our technical understanding of disparity measures (e.g., the choice of absolute or relative measures) may support such discussions [107].

A third overarching issue is the predominant focus on differences between groups defined by a single axis —mostly race and/or ethnicity—which fails to recognize the multiple dimensions of identity and disadvantage. As the diversity of nursing home residents broadens and increases in many parts of the world, the possible contribution of intersectionality in understanding health disparities in nursing homes deserves considered attention. Intersectionality encourages critical reflection to move beyond singular categories such as race or socioeconomic status [108] and recognize that these are not independent strands, but rather intersecting constructs of inequality [109]. When developing research questions and studies, researchers could consider intersectionality by carefully thinking about what social categories are relevant to the outcome, who is included (and thus excluded) in a social category, and how multiple social categories intersect and influence each other [110]. Furthermore, researchers could consider examining the broader historical and current social, economic, and political contexts that may influence the outcomes or experiences of the groups being studied, and engaging and collaborating with the communities being studied to ensure the research is relevant and respectful of their experiences [111, 112]. By integrating these considerations, researchers can develop more comprehensive and meaningful studies that address the multifaceted nature of health disparities.

Fourth, in addition to quantifying health disparities among nursing home residents, future work focusing on disparities in patient experience by leveraging patient-reported experience of care and/or patient satisfaction would be a novel avenue. Beyond secondary data analyses of administrative data, qualitative research or surveys can add to our understanding of how nursing home residents experience the care they receive by capturing individuals’ narratives, preferences, and expectations. Such knowledge can contribute to healthcare providers, researchers, and policymakers asking more meaningful questions, collecting data on more relevant outcomes, and designing interventions better tailored to address health disparities.

Fifth, future studies need greater clarity in articulating whether they are examining health disparities among residents within the same nursing homes, versus across the population of all persons who receive care in nursing homes [5]. This is important, since interventions to reduce disparities in care within a single nursing home are not necessarily the same as those needed to address socioeconomic and policy issues at the community or government level to mitigate disparities across the long-term care system.

This review aims to inspire and support researchers globally to engage in health disparities research. While much of the existing research originates from the U.S., health disparities are a worldwide concern. By sharing experiences and approaches, countries can enhance their research capacity, foster international collaboration, and improve the overall quality of health disparities research.

A major strength of U.S.-based disparities research is the availability of large, comprehensive secondary datasets which enables detailed quantitative and observational analyses. National healthcare systems vary in their progress towards integrating advanced data collection techniques, such as electronic health records and patient registries, to monitor and address health disparities across different regions. Furthermore, the extent of data collection on nursing home populations, nursing home facilities, community and policy-level factors, varies significantly across countries. This review may contribute to countries evaluating what data they need to collect and how to effectively study disparities in their nursing home population to help in developing targeted interventions to address specific health disparities. Additionally, implementing data-sharing initiatives, such as easily accessible centralized health data repositories, can lead to more efficient and equitable resource distribution, as well as fairness in access to data. These initiatives can also facilitate learning from each other by allowing countries to understand different approaches to data collection and analysis. This can help improve the overall quality and effectiveness of health disparities research globally.

## Methods discussion

In conducting this scoping review, several methodological considerations and potential limitations were identified. First, there is a risk of selection and publication bias as the concepts of health disparities and inequity are inconsistently indexed and operationalized, making it difficult to reliably identify all relevant studies [19].

Second, we observed that combining searches at the title and abstract screening stage, rather than at the full text screening stage, could streamline the process by reducing overlap earlier. The specific search strategy alone identified 90% of the included studies, demonstrating its efficiency in capturing relevant studies. Nevertheless, our assessment is that this approach has yielded valuable insights into the strengths and weaknesses of the individual search strategies, which will be beneficial for other researchers investigating health disparities literature.

Third, we only included peer-reviewed original research retrieved from databases and might have missed out on relevant evidence from dissertations, conference abstracts, grey literature, doing hand searches, and so on.

Fourth, we acknowledge that deciphering health disparity, in some cases, was more difficult than anticipated. Some papers, that aimed to describe a variety of factors associated with one or more outcomes, were excluded, which potentially led us to miss out on relevant data.

Fifth, the categorization of outcomes in this study was not straightforward, and some conceptual overlap exists between domains, and some categories could fit in multiple domains. This grouping was based on our professional judgment, and we have made every effort to be transparent and reproducible in our approach, clearly indicating which categories fall under which domains and how many studies are included in each category.

## Conclusion

Much knowledge has been gained about health disparities in nursing homes in recent years. Our scoping review provides valuable insights into the methods used in U.S. nursing home research to examine health disparities, offering guidance on how to set up and conduct disparities research.

Considerations for future research could include clearly defining and distinguishing between health disparities and health equity and progressing from simply describing disparities to uncovering the root causes behind them. Furthermore, expanding the scope of disparity analysis by considering the multiple dimensions of identity and disadvantage, and incorporating qualitative data to capture the experiences of health disparities. Lastly, it is important for future studies to clarify whether they are contributing to knowledge about variations within a single nursing home or across different nursing homes.

## Supplementary Information

Supplementary Material 1.

## Data Availability

No datasets were generated or analysed during the current study.
