# Supplementary material for: Methods for studying health disparities in U.S. nursing homes: a scoping review
Source: BMC Health Serv Res. 2025 Aug 20;25:1117. doi: 10.1186/s12913-025-13071-3 (PMC12369220; doi:10.1186/s12913-025-13071-3)
Supplement: Supplementary file 1 — Supplementary Material 1. [file 12913_2025_13071_MOESM1_ESM.docx]

**Supplementary file 1** Search strategy in Medline (Ovid)

| Specific aspect search | Nursing homes OR Nursing home* (kw) OR Homes for the aged OR Skilled nursing facilities OR Nursing facilit* (kw) OR Long-term care facilit* AND Minority health OR Minority groups OR Health Disparate, Minority and Vulnerable Populations OR Vulnerable populations OR Social determinants of health OR Race factors OR Racial groups OR Ethnic and racial minorities OR Ethnicity OR Socioeconomic factors OR Sex factors OR Gender identity  OR Sexual and gender minorities OR Indigenous peoples  OR Health services, indigenous OR Residence Characteristics OR Dementia OR Rural Health Services OR Rural Health OR Urban Health Services OR Urban Health  AND limit to English language |
| --- | --- |
| Broad concept search | Nursing homes OR Nursing home* (kw) OR Homes for the aged OR Skilled nursing facilities OR Nursing facilit* (kw) OR Long-term care facilit* AND Healthcare disparities OR Healthcare disparit* OR Disparity (kw) OR  Disparities (kw) OR Health status disparities OR Health OR status disparit* (kw) OR Health inequities OR Health inequit* (kw) OR Inequit* (kw) OR Health services accessibility OR Health service* access* (kw) AND limit to English language |

We used subject terms and keywords to capture articles not yet assigned subject terms, but did not conduct separate searches on entry terms. Instead, we used subject terms with explode to capture narrower terms.
